# Conversion of cellulosic materials into glycolipid biosurfactants, mannosylerythritol lipids, by *Pseudozyma* spp. under SHF and SSF processes

**DOI:** 10.1186/s12934-014-0155-7

**Published:** 2014-11-04

**Authors:** Nuno Torres Faria, Marisa Santos, Carla Ferreira, Susana Marques, Frederico Castelo Ferreira, César Fonseca

**Affiliations:** Department of Bioengineering and IBB - Institute for Bioengineering and Bioscience, Instituto Superior Técnico, Universidade de Lisboa, Av. Rovisco Pais, 1049-001 Lisboa, Portugal; MIT-Portugal Program, 77 Massachusetts Avenue, E40-221, Cambridge, MA 02139 USA; Laboratório Nacional de Energia e Geologia, I.P, Unidade de Bioenergia, Estrada do Paço do Lumiar 22, 1649-038 Lisboa, Portugal

**Keywords:** *Pseudozyma* spp, Yeasts, Cellulosic materials, Wheat straw, Glycolipids, Mannosylerythritol lipids, Biosurfactants, Cellulolytic enzymes, SHF, SSF

## Abstract

**Background:**

Mannosylerythritol lipids (MEL) are glycolipids with unique biosurfactant properties and are produced by *Pseudozyma* spp. from different substrates, preferably vegetable oils, but also sugars, glycerol or hydrocarbons. However, solvent intensive downstream processing and the relatively high prices of raw materials currently used for MEL production are drawbacks in its sustainable commercial deployment. The present work aims to demonstrate MEL production from cellulosic materials and investigate the requirements and consequences of combining commercial cellulolytic enzymes and *Pseudozyma* spp. under separate hydrolysis and fermentation (SHF) and simultaneous saccharification and fermentation (SSF) processes.

**Results:**

MEL was produced from cellulosic substrates, Avicel® as reference (>99% cellulose) and hydrothermally pretreated wheat straw, using commercial cellulolytic enzymes (Celluclast 1.5 L® and Novozyme 188®) and *Pseudozyma antarctica* PYCC 5048^T^ or *Pseudozyma aphidis* PYCC 5535^T^. The strategies included SHF, SSF and fed-batch SSF with pre-hydrolysis. While SSF was isothermal at 28°C, in SHF and fed-batch SSF, yeast fermentation was preceded by an enzymatic (pre-)hydrolysis step at 50°C for 48 h. *Pseudozyma antarctica* showed the highest MEL yields from both cellulosic substrates, reaching titres of 4.0 and 1.4 g/l by SHF of Avicel® and wheat straw (40 g/l glucan), respectively, using enzymes at low dosage (3.6 and 8.5 FPU/g_glucan_ at 28°C and 50°C, respectively) with prior dialysis. Higher MEL titres were obtained by fed-batch SSF with pre-hydrolysis, reaching 4.5 and 2.5 g/l from Avicel® and wheat straw (80 g/l glucan), respectively.

**Conclusions:**

This work reports for the first time MEL production from cellulosic materials. The process was successfully performed through SHF, SSF or Fed-batch SSF, requiring, for maximal performance, dialysed commercial cellulolytic enzymes. The use of inexpensive lignocellulosic substrates associated to straightforward downstream processing from sugary broths is expected to have a great impact in the economy of MEL production for the biosurfactant market, inasmuch as low enzyme dosage is sufficient for good systems performance.

## Background

Surfactants are amphiphilic molecules possessing surface and interfacial activity and the ability of forming organized molecular assemblies, monolayers, micelles, vesicles and membranes [1,2]. Biosurfactants are produced by a variety of microorganisms including bacteria, filamentous fungi and yeasts from different substrates, including oils, sugars and glycerol. Biosurfactants are a group of bio-based products with increasing scientific, environmental and economic interest. These bio-based products are expected to partially replace conventional (oil-based) surfactants and, due to their unique structures, properties, low toxicity and high biodegradability, may conduct to novel applications in industrial and environmental biotechnology sectors [1,2].

**Figure 1:**
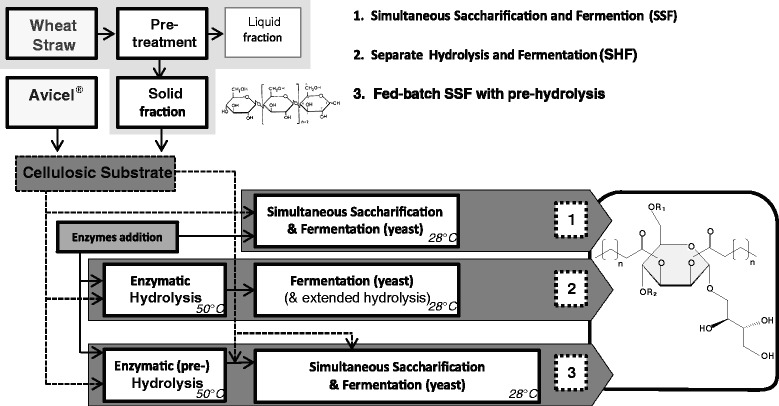
**Strategies for MEL production from cellulosic substrates (Avicel** ^**®**^ **and solid fraction of pretreated wheat straw) combining enzymatic hydrolysis and fermentation with** ***Pseudozyma*** **spp.** 1. Simultaneous saccharification and fermentation (SSF), at 28°C; 2. Separate hydrolysis and fermentation (SHF), with an enzymatic hydrolysis step at 50°C, for 48 h, and a fermentation step at 28°C; 3. Fed-batch SSF, with a (pre-)hydrolysis step at 50°C, for 48 h, followed by fermentation (and enzymatic hydrolysis) with pulsed feeding of substrate, at 28°C. Structure of MEL: MEL-A: R1 = R2 = Ac; MEL-B: R1 = Ac, R2 = H; MEL-C: R1 = H, R2 = Ac; Acyl groups: 8–12 carbons (n = 6-10).

Among microbial surfactants, the glycolipids known as mannosylerythritol lipids (MEL) (Figure 1) contain 4-O-β-D-mannopyranosyl-meso-erythritol and fatty acids as the hydrophilic and hydrophobic moieties, respectively [2,3]. According with the degree of acetylation at mannosyl C-4 and C-6, and their elution on thin-layer chromatography (TLC), MELs are classified as MEL-A, -B, -C and -D. MEL-A represents the diacetylated compound while MEL-B and MEL-C are monoacetylated at mannosyl C-6 and C-4, respectively. The completely deacetylated structure is known as MEL-D [3]. The sugar moiety is acylated at mannosyl C-2 and C-3 with heterogeneous fatty acid chains usually containing 8 to 12 carbons [3].

MELs exhibit excellent surface-active properties and versatile biochemical functions including: antimicrobial activity against gram positive bacteria [4]; excellent growth inhibition and differentiation-inducing activities against human leukemia cells [5], rat pheochromocytoma [6] and mouse melanoma cells [7]; high affinity binding towards different immunoglobulins [8] and lectins [9]; properties of self-assembling [10,11], with potential use in gene transfection and drug delivery [12]; constituent of ice-slurry as an anti-agglomeration compounds [13]; skin care properties by recovery effect on SDS-induced damaged cells treated with MEL-A, suggesting MEL-A has ceramide-like skin care properties [14].

MEL is mainly produced by anamorphic basidiomycetous yeasts *Pseudozyma* spp. and fungi, *Ustilago maydis* [1,2]. Several substrates have been used for MEL production with *Pseudozyma* spp., including soybean oil, alkanes, glycerol, glucose and xylose [3,15-19]. The metabolism of *Pseudozyma* spp. for MEL production from sugar and oils is necessarily different. The utilization of lipidic substrates by yeasts requires active lipid metabolism, including the action of lipases, activation of fatty acids, β-oxidation towards the production of shorter fatty acids and C2 units, which in turn are used in gluconeogenesis for the production of sugar-phosphates. The carbohydrate metabolism involves glycolysis, pentose phosphate pathway (PPP) and fatty acids biosynthesis, with high demand for NADPH. Soybean oil has been the preferred substrate for MEL production, leading to the highest titres, yields and productivities, probably because the lipidic moiety is directly generated by partial β-oxidation of oil fatty acids and does not require *de novo* synthesis [20].

However, the sustainability of MEL production from lipidic substrates is doubtful due to the environmental impact of cultivation of dedicated crops for oil production, to the price of vegetable oils, and to the difficulties associated with MEL recovery from oily broths, which requires solvent intensive and low-yield downstream separation processes [2,21]. Therefore, the use of sugar substrates for MEL production may represent an upgrading on process sustainability, both on substrate level, if using inexpensive substrates with low environmental impact (like lignocellulosic residues), and on downstream level, due to the requirement of a single solvent extraction process for MEL recovery.

Lignocellulosic materials are the focus of relevant scientific and industrial efforts that are being made to build efficient and sustainable technologies for the conversion of renewable carbon sources into advanced biofuels and other bio-based products [22-24]. Lignocellulose, the most abundant renewable carbon resource on earth, is present in wood, agricultural and forest residues, agro-industrial and municipal solid wastes, and is mainly composed of cellulose (40-60%), hemicellulose (20-40%) and lignin (15-30%) [23,25].

The development of efficient technologies for the conversion of lignocellulosic materials into ethanol is enabling the production of other bio-based products with a potential positive impact in the environment and in the expansion of the bioeconomy [22,24]. Lignocellulosic ethanol usually includes a physical-chemical pretreatment of biomass, followed by enzymatic hydrolysis of the cellulose fraction and fermentation of released sugars by yeasts (*Saccharomyces cerevisiae*) [25]. Enzymatic hydrolysis and fermentation may occur sequentially as separate hydrolysis and fermentation (SHF) or at the same time, as simultaneous saccharification and fermentation (SSF). The SSF process significantly decreases product inhibition of cellulolytic enzymes since the sugars released during hydrolysis are continuously consumed by the fermenting microorganism [26]. In both processes, enzymes represent one of the most significant operational costs of lignocellulosic ethanol. Therefore the use of low enzyme loading is desirable, even if significant improvements have been achieved on enzyme engineering and reduction on production cost [26]. An example is the engineering of β-glucosidases towards the alleviation of end-product inhibition, which has been one of the focuses of research by enzyme manufacturers in the development of superior commercial enzyme cocktails [27], allowing SHF processes to be more efficient. Although some microorganisms are able, or have been engineered, to assimilate pentoses and/or produce their own cellulolytic and hemicellulolytic enzymes enabling Consolidated Bioprocessing (CBP), most commercial or pre-commercial processes for the production of biofuels [25], chemicals (e.g. succinic acid) [28] and materials (e.g. PHA) [29] still operate under SHF or SSF using only the cellulosic fraction of lignocellulosic materials [30].

The present study aims to demonstrate, for the first time, the conversion of cellulosic materials into glycolipid biosurfactants, MEL, using a model substrate, Avicel® cellulose, and a natural lignocellulosic substrate, pretreated wheat straw (cellulose-enriched solid fraction). While Avicel® is commercial crystalline cellulose (>99%), lignocellulosic residues, like forestry, agro-industrial and agriculture residues (e.g. wheat straw), often contain approx. 50% (on a dry-weight basis) of cellulose [31] and, after hydrothermal pretreatment (autohydrolysis), the solid fraction is enriched in cellulose and lignin due to the solubilisation of hemicellulose. The biochemical conversion of the two cellulosic materials into MEL was investigated by the combination of commercial cellulolytic enzyme cocktails and two different *Pseudozyma* strains under different process configurations (SHF, SSF and Fed-batch SSF with pre-hydrolysis).

## Results and discussion

### Overview of strategies for MEL production from cellulosic materials

The aim of the current study was to investigate the use of cellulose-rich substrates, represented by the solid fraction of pretreated wheat straw (composition presented in Table 1), for the production of MEL by *Pseudozyma* yeasts, *P. antarctica* PYCC 5048^T^ and *P. aphidis* PYCC 5535^T^. Avicel® was used as reference cellulosic substrate since it consists of pure crystalline cellulose. The overall strategy considered the development of different process configurations with the use of commercial cellulolytic enzyme cocktails to support the saccharification of cellulose prior to yeast bioconversion of glucose into MELs (Figure 1). First, this work explored two strategies: simultaneous saccharification and fermentation (SSF) and separate hydrolysis and fermentation (SHF). The SSF process was performed at 28°C, which according with previous studies is within the optimal temperature range for *Pseudozyma* spp. growth and MEL production [3,32]. Under SSF, glucose released by enzymatic hydrolysis was concomitantly consumed by yeast. The SHF setup consisted of a two-step process, with an enzymatic hydrolysis step at the optimal temperature of commercial cellulolytic enzymes, 50°C [33], for 48 h, followed by yeast bioconversion at 28°C for 10 days. In order to improve MEL titres, a third approach, fed-batch SSF with enzymatic pre-hydrolysis, was performed.

**Table 1:** **Lignocellulosic composition of the solid fraction of pretreated wheat straw**

**Lignocellulosic component**	**Content (g/100 g dry matter)**
Glucan (cellulose)	59.0
Xylan (hemicellulose)	11.3
Lignin	21.7

The use of a separate hydrolysis step has the potential of making available a significant fraction of fermentable sugars in the beginning of the yeast bioconversion process. Preliminary studies revealed that glucose release from 4.0% w/v Avicel® increased with enzyme loading, with 50% or 71% of total cellulose converted into glucose at low or medium enzyme dosage, respectively, after a 48 h-hydrolysis at 50°C. In the SHF strategy followed in this study, enzymes were not removed before fermentation and, therefore, after the enzymatic hydrolysis stage at 50°C, hydrolysis of the remaining cellulose continues during *Pseudozyma* spp. conversion of sugars into MEL, at yeast optimal growth temperature, i.e. 28°C. However, enzymatic hydrolysis at 28°C is slower than at 50°C for the same enzyme loading, which is explained by lower cellulolytic activities at 28°C than at 50°C (40% and 20% activity at 28°C for Celluclast 1.5 L® and Novozyme 188®, respectively).

The availability of sugars obtained from enzymatic hydrolysis influences the bioconversion process, as in SSF processes for the production of ethanol with *Saccharomyces cerevisiae*, where fermentation is often limited by the rate of hydrolysis, i.e. glucose concentration is virtually zero throughout the process [34]. However, the specific glucose consumption rate of *Pseudozyma* spp. is lower than *S. cerevisiae*. Therefore, enzyme loading and consequent rate of cellulose enzymatic hydrolysis can be balanced with yeast loading and respective glucose consumption rate.

Under this perspective, the kinetics of glucose release by enzymatic hydrolysis and glucose consumption by yeast were assessed at 28°C, towards a SSF approach. Enzymatic hydrolysis of 4.0% w/v Avicel® at different enzyme loading (low, medium and high, i.e. 3.6, 10 and 30 FPU/g, respectively) generated glucose at average rates of 0.2-0.3 g/l/h, at 28°C, during the first 96 h. In parallel, glucose consumption rate by *P. antarctica* and *P. aphidis*, under favourable conditions for MEL production (28°C, approx. 0.5 g/L yeast loading), was 0.18 and 0.21 g/l/h, respectively. Under these consumption rates, SSF process can be performed at low enzyme loading, with benefits in the economy of MEL production from cellulosic materials. The influence of process configuration (SHF vs SSF), substrate (Avicel® vs wheat straw), enzyme loading and feeding strategy were evaluated.

### SSF of Avicel®

**Figure 2:**
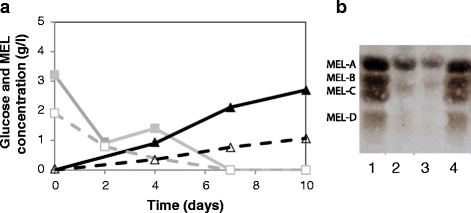
**MEL production from commercial grade cellulose (Avicel** ^**®**^ **) by** ***P. antarctica*** **PYCC 5048** ^**T**^ **and** ***P. aphidis*** **PYCC 5535** ^**T**^ **by SSF.** SSF was carried out with 40 g/l glucan at 28°C and 140 rpm, supplemented with low enzyme loading. **(a)** Time course of SSF showing glucose concentration (squares) and MEL formation (triangles) with *P. antarctica* (continuous line) and *P. aphidis* (dashed line). **(b)** Thin Layer Chromatography (TLC) of MEL obtained from liquid-liquid extraction with ethyl acetate of culture broth samples (after 10 days of SSF). Samples were eluted with the solvent system of CHCl3/CH3OH/NH4OH (65:15:2) to separate MEL-A, -B, -C and -D, which were visualized after reaction of α-naphthol reagent with the sugar moiety of MEL. Lines: 1 and 4 - MEL standard (MEL-A, -B, -C and -D [19]); 2 - *P. antarctica* SSF; 3 - *P. aphidis* SSF.

MEL production was initially investigated in SSF experiments using Avicel®, a model substrate consisting of pure crystalline cellulose. The conversion of 4.0% w/v Avicel® was performed with low enzyme loading (3.6 FPU/g_glucan_ at 28°C) and approx. 0.5 g/l yeast loading (*P. antarctica* or *P. aphidis*). Both yeast strains consumed glucose concomitantly with sugar release from enzymatic hydrolysis, as denoted by vestigial glucose concentrations (<3 g/l) throughout SSF process (Figure 2a). This observation confirmed that enzymatic hydrolysis and glucose consumption rates under this SSF setup were similar. Glucose concentration decreased to virtually zero after day 4 and MEL accumulated up to 2.7 and 1.1 g/l with *P. antarctica* and *P. aphidis*, respectively.

When glucose is directly used as substrate (40 g/l) under similar conditions (media, carbon source concentration, temperature, agitation), the same strains generated maximum MEL titres of 5.4 g/l in *P. antarctica* and 3.4 g/l in *P. aphidis* [19], which are 2- and 3-fold higher than those obtained under SSF of Avicel®, respectively. In other study [29], other *P. antarctica* strain (T-34) and the same *P. aphidis* strain (PYCC 5535^T^, CBS 6821) generated 4.2 and 0 g/l of MEL from D-glucose, respectively. The reasons for the lower MEL titres and yields obtained in SSF of Avicel® when compared to those obtained in glucose cultivations were investigated in the next sections with respect to the influence of using commercial cellulolytic cocktails and to the initial glucose concentration (SSF vs SHF).

MEL production was confirmed by TLC (Figure 2b) using a MEL-A to -D reference. Glycolipid spots were identified in samples from both cultures at day 10, MEL-A being the most abundant MEL form.

**Figure 3:**
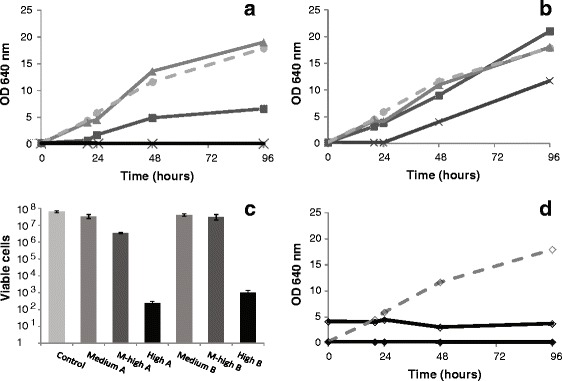
**Influence of commercial cellulolytic enzyme cocktails in yeast (** ***P. aphidis*** **) growth and viability in 40 g/l glucose (carbon source). (a)** Yeast growth in the presence of Celluclast 1.5 L® loading: medium, 1.75% v/v (triangles), medium-high, 3.50% v/v, (squares), high, 5.25% v/v (crosses); **(b)** Yeast growth in the presence of Novozyme 188® loading: medium, 0.25% v/v (triangles), medium-high, 0.50% v/v (squares), high 0.75% v/v (crosses); **(c)** Viable yeast cells after 96 h-culture in the presence of: medium (1.75% v/v), medium-high (3.5% v/v) and high (5.25% v/v) Celluclast 1.5 L® loading (A); and medium (0.25% v/v), medium-high, (0.50% v/v) and high (0.75% v/v) Novozyme 188® loading (B); **(d)** Yeast growth in the presence of Celluclast® 1.5 L, 5.25% v/v plus Novozyme 188®, 0.75% v/v (black lines): active enzymes (filled diamonds), inactivated enzymes (open diamonds). Control (glucose without enzymes) (dashed grey line, open diamonds).

The total fatty acid profile of the whole broth shows that MEL acyl groups (C8-C12) were mainly composed of C10:n and C12:n (Table 2). Such profile was slightly different from that found in MEL produced from soybean oil, preferentially C8:0 and C10:n, as previously reported [3]. The carbon chain length pattern of MEL acyl groups was similar to that found when sugars (hexoses and pentoses) or fatty acid methyl ester C18:0 were used as substrates [3,19]. This suggests that the building blocks of MEL lipidic chains are obtained from a partial β-oxidation of fatty acids, usually accumulated in the form of triglycerides (C16-C18) [20,35]. Accordingly, with this putative metabolic pathway, when Avicel® cellulose is used as substrate, sugars made available for cultivation are partially converted into fatty acids (mainly C18:n) and then, after an incomplete β-oxidation, the shorter acyl groups (C8-C12) are assembled to the sugar moiety (mannosylerythritol) for MEL production [20,36]. The metabolic pathways for MEL production from sugars are not yet fully understood, but the hypothesis presented above might explain why *P. antarctica* accumulated more C8-C12 fatty acids while *P. aphidis* accumulated more C16-C18 fatty acids (Table 2, Figure 3a), i.e. *P. antarctica* had a more efficient β-oxidation of C16-C18 fatty acids into C8-C12 (for MEL assembly) than *P. aphidis*, leading to relatively higher MEL titres in *P. antarctica* cultures. The lower MEL yield from glucose or cellulose than from lipids is probably related to the *de novo* fatty acids biosynthesis (C16-C18) and its energy requirements, which can be provided by NADPH generated through oxidative PPP at the expense of carbon.

**Table 2:** **Total fatty acid profile of the whole broth after 10 days of SSF of Avicel** **®**

**Fatty acid form**	***P. antarctica*** **PYCC 5048** ^**T**^		***P. aphidis*** **PYCC 5535** ^**T**^	
	**Concentration (mM)**	**Relative composition in MEL (%)**	**Concentration (mM)**	**Relative composition in MEL (%)**
C8:0	1.20	17.1	0.51	18.3
C10:n	3.64	51.8	1.32	47.5
C12:n	2.19	31.2	0.95	34.2
C14:0	0.00		0.37	
C16:0	0.96		6.40	
C18:n	2.90		12.67

### Selection of conditions for SSF and SHF processes

As in other bioprocesses, the conversion of cellulose into MEL can be accelerated by increasing the concentration, and thus the activity, of biocatalysts, in this case, enzyme and yeast. Moreover, increase in rate of enzymatic hydrolysis (i.e. the rate of glucose release from Avicel®) with temperature can be explored in SHF processes, where enzymatic hydrolysis can be performed at optimal temperature (50°C) prior to yeast inoculum. In this section, the advantages and disadvantages of increasing enzyme loading (Celluclast 1.5 L® and/or Novozyme 188®), as well as of using of different process configurations (SSF vs SHF), were evaluated.

#### Impact of enzyme dosage in yeast cultivation

Although the increase in enzyme loading has the potential of improving hydrolysis productivity, commercial cellulolytic enzymes may have a negative impact on cell viability and growth of (non-conventional) yeasts under SSF processes [37]. Therefore, growth and viability of yeasts were accessed under different enzyme loadings, using the same initial glucose content as carbon source (Figure 3). The commercial cellulolytic enzymes used in the cocktails were tested separately at low (L), medium (M), medium-high (MH) and high (H) loadings, i.e. concentration ranging from 1.75 to 5.25% v/v of Celluclast 1.5 L® (Figure 3a and 3c – A) or from 0.25 to 0.75% v/v of Novozyme 188® (Figure 3b and 3c – B) (see details in Materials and methods section). Growth rate (Figure 3a) and viability (Figure 3c – A) were significantly reduced at high Celluclast 1.5 L® loading, moderately affected at medium-high enzyme dosage, but the effects observed were negligible at medium enzyme loading. In the case of Novozyme 188®, only at high enzyme dosage (0.75% v/v) yeast viability (Figure 3c – B) was significantly reduced and growth (Figure 3b) was moderately affected. These results recommend the use of low or medium enzyme loading in SHF and SSF processes, where growth and relatively long yeast cultivation periods are expected to occur after or during enzymatic hydrolysis. The combination of both commercial enzymes in cocktails at low (0.58% v/v Celluclast 1.5 L® and 0.08% v/v Novozyme 188®) and medium (1.75% v/v Celluclast 1.5 L® and 0.25% v/v Novozyme 188®) loading did not affect growth (data not shown), indicating no negative synergistic effects at low and medium cocktail loadings.

The negative effect of Celluclast 1.5 L® on cell viability and growth rate was previously reported for the yeast *Kluyveromyces marxianus* [37]. However, in that case, ethanol production was not significantly reduced. Here, the impact of the combination of both commercial enzymes in MEL production was evaluated at medium and high loading, using glucose as carbon source (Table 3). High loading of enzyme cocktail impaired MEL production from glucose by both *P. antarctica* and *P. aphidis*. Although medium enzyme loading did not affect yeast growth, this enzyme dosage reduced MEL production in 60-70% in comparison to the control (glucose, enzyme not applied) (Table 3).

**Table 3:** **Effect of enzyme loading in MEL production by** ***P. antarctica*** **PYCC 5048** ^**T**^ **and** ***P. aphidis*** **PYCC 5535** ^**T**^ **using glucose as carbon source, after 14 days at 28°C, 140 rpm**

**Enzyme loading**	**MEL (g/l)**	
	***P. antarctica*** **PYCC 5048** ^**T**^	***P. aphidis*** **PYCC 5535** ^**T**^
n.a.^a^	3.4 ± 0.2	2.3 ± 0.1
Medium^b^	1.1 ± 0.1	0.9 ± 0.3
High^c^	0.0 ± 0.0	0.0 ± 0.0

^a^Not applied.

^b^Celluclast 1.5 L® (1.75% v/v) and Novozyme 188® (0.25% v/v).

^c^Celluclast 1.5 L® (5.25% v/v) and Novozyme 188® (0.75% v/v).

To understand if the negative effect on cell viability, growth rate and MEL production resulted from the direct action of the hydrolytic enzymes present in the cocktail, those were inactivated at 100°C for 10 min, prior to use. The use of inactivated enzymes still negatively affected growth (Figure 3d), which points out for an inhibitory effect promoted by other cocktail component(s), possibly enzyme preservatives or stabilizers, rather than by enzyme activity. Therefore, the solutions of enzyme cocktails went through dialysis prior to use in order to remove putative inhibitors of yeast growth and metabolism present in commercial enzyme cocktails. Dialysis increased MEL production both for SHF and SSF process as discussed below.

#### Comparison of the system performance under SHF and SSF processes

The SSF process was performed isothermally at 28°C, which is within the range of optimal temperature for *Pseudozyma* spp. growth and metabolism towards maximum MEL production [3,32], but sub-optimal for enzymatic hydrolysis [34]. However, to enhance enzymatic hydrolysis and understand if the higher sugar concentration at the beginning of the cultivation promotes higher MEL production, the SHF process was also performed, with enzymatic hydrolysis taking place at 50°C for 48 h, followed by yeast loading and fermentation (and further enzymatic hydrolysis) at 28°C for, at least, 10 days.

MEL production was assessed in SHF and SSF processes with 4.0% w/v Avicel^®^ at low enzyme loading with and without prior dialysis. Interestingly, *P. antarctica* generated higher MEL titres (max. 4.0 g/l) under SHF, while *P. aphidis* generated higher MEL titres (max. 1.9 g/l) under SSF, both with prior enzyme dialysis (Table 4). This differential behaviour is in line with the hypothesis that *P. antarctica* is not limited in the step of partial β-oxidation of fatty acids, and thus can benefit from higher initial glucose concentration (generated in the hydrolysis step of SHF) and consequent higher intracellular fluxes for the production of fatty acids.

**Table 4:** **MEL production (titres and yields) and initial glucose concentration for SSF and SHF of Avicel** **®** **and wheat straw, using** ***P. antarctica*** **PYCC 5048** ^**T**^ **and** ***P. aphidis*** **PYCC 5535** ^**T**^ **and low enzyme loading (with prior dialysis)**

	***P. antarctica*** **PYCC 5048** ^**T**^			***P. aphidis*** **PYCC 5535** ^**T**^		
	**Initial glucose concentration (at fermentation step)** ^**b**^ **(g/l)**	**MEL (g/l)**	**Y**_**MEL/S**_^**c**^ **(g/g)**	**Initial glucose concentration (at fermentation step)** ^**b**^ **(g/l)**	**MEL (g/l)**	**Y**_**MEL/S**_^**c**^ **(g/g)**
**Avicel®**						
SSF^a^	< 3.0	2.9 ± 0.0	0.07	< 3.0	1.9 ± 0.1	0.05
SHF^b^	23.8 ± 1.9	4.0 ± 0.3	0.10	25.4 ± 1.1	1.1 ± 0.3	0.03
**Wheat straw**						
SSF^a^	< 3.0	1.1 ± 0.3	0.03	< 3.0	0.9 ± 0.4	0.02
SHF^b^	25.6 ± 1.0	1.4 ± 0.2	0.04	23.8 ± 1.1	0.9 ± 0.1	0.02

^a^SSF: enzymatic hydrolysis and fermentation were carried simultaneously with 40 g/l glucan, at pH 5.5, 50°C, 140 rpm for 10 days.

^b^SHF: enzymatic hydrolysis was carried out at 40 g/l glucan, pH 5.5, 50°C, 140 rpm for 48 h; fermentation (and further enzymatic hydrolysis) were conducted at pH 5.5, 28°C, 140 rpm for 10 days.

^c^MEL produced (g) per total glucan (g).

Dialysis of enzyme cocktails promoted an improvement in MEL production from 4.0% w/v Avicel®, both under SHF and SSF processes. The effect of dialysis was more pronounced in *P. aphidis* than in *P. antarctica*, with an increase in MEL production over 80% under both processes. In *P. antarctica* the improvement in MEL titres was 11% under SSF and 41% under SHF. These results indicate that dialysis is required to remove inhibitors of yeast metabolism and to improve MEL production.

The use of dialysed medium enzyme dosages in SHF and SSF processes generated similar MEL concentrations to those obtained with dialysed low enzyme dosages (data not shown). Therefore, dialysed low enzyme loading were used in subsequent studies, inasmuch as enzymes are one of the most important costs in both SSF and SHF processes [20] and the optimization towards use of low enzyme loading is critical for cost-effective MEL production from lignocellulosic biomass.

**Figure 4:**
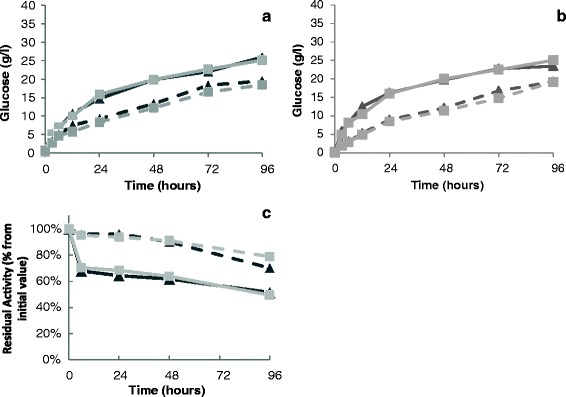
**Effect of dialysis on the performance of cellulolytic commercial enzyme cocktails on the cellulosic substrates used.** Kinetics of enzymatic hydrolysis of Avicel® **(a)** and pretreated wheat **(b)** straw and enzyme thermal stability **(c)** were performed at 50°C (continuous lines) and 28°C (dashed lines) during 96 h, at low enzyme loading with (dark grey) or without (light grey) prior dialysis.

Furthermore, the kinetics of enzymatic hydrolysis and enzyme stability were characterized with low enzyme loading (with and without dialysis), at 28°C and 50°C, using Avicel^®^ (4.0% w/v) as substrate. The dialysis of the enzyme cocktail did not affect enzyme activity since similar rates and yields of hydrolysis were achieved with dialysed and non-dialysed enzyme cocktails at both 28°C and 50°C (Figure 4a,b). In agreement, enzyme stability over time was similar with or without dialysis of enzyme cocktails (Figure 4c). Noteworthy, at 28°C, the enzyme activity was kept above 70% over the 4 days of experiment and over 90% of the initial enzymatic activity for at least 48 h (from 3.6 FPU/g to 3.2 FPU/g) (see Figure 4c). However, although enzymatic hydrolysis is more efficient at 50°C (Figure 4a), a steeply loss of enzyme activity to values around 60-70% was observed after 6 h (from 8.5 FPU/g to 5–6 FPU/g) (Figure 4c), reaching a value of 50% by day 4. Therefore, in SHF, the enzymatic hydrolysis at 50°C during 48 h, with low enzyme dosage, generated a 50% glucan conversion into glucose after 48 h, still maintaining more than 60% of enzyme activity, while in SSF (isothermal process at 28°C), the glucose release was slower but enzyme activity was maintained close to maximum activity for the first 48 h. Although Celluclast 1.5 L® is relatively stable at 50°C, Novozyme 188® (β-glucosidase) suffers significant heat-induced denaturation at this temperature leading to protein precipitation, which in the case of Celluclast 1.5 L®/Novozyme 188® cocktails correspond to 50% after 4-days incubation [38].

### Comparison of SSF and SHF of Avicel® and wheat straw for MEL production under selected conditions

Both SHF and SSF processes were applied for MEL production with wheat straw using *P. antarctica* or *P. aphidis* and dialysed low enzyme loadings and compared with the results obtained with Avicel®. Wheat straw went through prior hydrothermal pretreatment, which optimal conditions where previously established for maximal sugar recovery [39]. The solid fraction obtained, mainly composed of cellulose and lignin (Table 1), was used as substrate for MEL production from natural lignocellulosic materials, at 6.6% w/v solids, i.e. at 40 g/l glucan, and the results were compared to those with 4.0% w/v Avicel® (Table 4), also at 40 g/l glucan.

The kinetics of enzymatic hydrolysis of the solid fraction of pretreated wheat straw (6.6% w/v) was similar either using dialysed or non-dialysed enzyme cocktails and to that of 4.0% w/v Avicel® (Figure 4a,b). By washing the solids after hydrothermal pretreatment, the potential inhibitors of enzymatic hydrolysis (and fermentation) were removed, producing no effect on enzymatic hydrolysis performance when compared to the hydrolysis of Avicel®. Also, under those conditions, the presence of lignin and other constituents of pretreated wheat straw did not significantly affect enzyme performance when compared to the hydrolysis of pure (crystalline) cellulose (Avicel®), even if exhibiting higher viscosity at the same glucan content. Accordingly, under SHF, the enzymatic hydrolysis step (48 h at 50°C) released 23–25 g/l of glucose from both Avicel® and wheat straw (Figure 4a,b, Table 4).

After yeast inoculation (10% v/v inoculum), glucose concentration was reduced to low levels between days 4 and 7 (<4 g/l) and became not detectable at day 10. In SHF of wheat straw, xylose (<3 g/l) was obtained from the hydrolysis of hemicellulose present in the solid fraction and maintained up till day 7, becoming virtually zero at day 10, which confirmed xylose assimilation capacity by *Pseudozyma* spp. [19]. The highest MEL production was obtained using Avicel® and *P. antarctica* in the SHF approach resulting in a MEL titre of 4.0 g/l and yield of 0.10 g_MEL_/g_glucan_. *Pseudozyma antarctica* and *P. aphidis* produced MEL from SHF of wheat straw at concentrations of 1.4 and 0.9 g/l, respectively, after 10 days (Table 4).

In the SSF processes, the initial sugar concentration (glucose and xylose) did not exceed 3 g/l, remaining low until day 7, and became virtually zero at day 10. Using wheat straw as substrate, both *P. antarctica* and *P. aphidis* produced MEL at 1.1 and 0.9 g/l, respectively, after 10 days. Those values are lower than when Avicel® was used as substrate for SSF, with *P. antarctica* reaching 2.9 g/l and *P. aphidis* 1.9 g/l of MEL. SHF and SSF processes using wheat straw as carbon source generated similar MEL yields of 0.03-0.04 g_MEL_/g_glucan_ with *P. antarctica* and 0.02 g_MEL_/g_glucan_ with *P. aphidis*. The fatty acid profile of *P. antarctica* and *P. aphidis* cultures in the bioconversion of wheat straw revealed MEL acyl groups mainly composed of C10:n and C12:n (data not shown), which was similar to the profile obtained from Avicel® (see Table 2) and glucose cultures [16,19]. In summary, *P. antarctica* produced significantly (p < 0.05) higher MEL titres under SHF than under SSF for both substrates (approx. 40% more with Avicel^©^ and 30% more with pretreated wheat straw). In turn, MEL obtained from Avicel® was significantly (p < 0.05) higher (3-fold) than that from wheat straw. On contrary, *P. aphidis* produced more MEL under SSF than under SHF of Avicel®, but not with wheat straw.

The main difference between the two cellulosic substrates set up was related to the total amount of solids in order to meet the same glucan content (40 g/l). With wheat straw, a total amount of 6.6% (w/v) solids is required due to the presence of lignin. Taking into account that the presence of lignin had no significant impact on enzymatic hydrolysis when comparing glucose yield from wheat straw and Avicel® (see Figure 4), lignin might have had a negative impact on yeast bioconversion due to potential chemical and/or physical interaction, namely by affecting the rheology of the system as a result of the higher viscosity. This effect of lignin was significant for *P. antarctica* under both configurations (SHF and SSF) but only significant for *P. aphidis* under SSF.

Taking into account that *P. antarctica* generated the highest MEL yields both from Avicel® and wheat straw, this strain was selected for fed-batch studies in the conversion of cellulosic materials into MEL aiming at increasing MEL titres.

### Fed-batch SSF strategies with pre-hydrolysis

**Figure 5:**
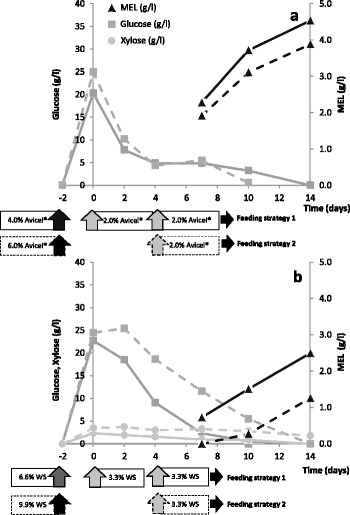
**MEL production from Avicel® (a) and wheat straw (WS) (b) by fed-batch SSF (at 28°C) with (pre-)hydrolysis (at 50°C) using** ***P. antarctica*** **PYCC 5048** ^**T**^ **and low enzyme loading with prior dialysis.** D-Glucose (squares), D-xylose (circles) and MEL (triangles) concentrations. Feeding strategy 1 (continuous lines): initial 40 g/l glucan content (4.0% w/v Avicel® or 6.6% w/v wheat straw) for pre-hydrolysis (48 h) and further 20 g/l glucan feeding (2.0% w/v Avicel® or 3.3% w/v wheat straw) at day 0 and day 4. Feeding strategy 2 (dashed lines): initial 60 g/l glucan content (6.0% w/v Avicel® or 9.9% w/v WS) for pre-hydrolysis (48 h) and further 20 g/l glucan feeding (2.0% w/v Avicel® or 3.3% w/v WS) at day 4.

In order to improve MEL titres from cellulosic materials, a fed-batch SSF with pre-hydrolysis approach was used, where the enzymatic pre-hydrolysis was performed at 50°C for 48 h, followed by yeast loading, fermentation at 28°C and substrate feeding during the process. The goal was to increase total substrate loading, up to approx. 80 g/l glucan, either using Avicel® (8.0% w/v) or pretreated wheat straw (13.2% w/v). Two different strategies of fed-batch SSF with pre-hydrolysis were used (Figure 5): Feeding strategy 1 – initial (day −2) 40 g/l glucan (4.0% w/v Avicel® or 6.6% w/v wheat straw) for 48 h pre-hydrolysis and two pulses of 20 g/l glucan (2.0% w/v Avicel® or 3.3% w/v wheat straw) at day 0 and day 4; Feeding strategy 2 - initial (day −2) 60 g/l glucan (6.0% w/v Avicel® or 9.9% w/v wheat straw) for 48 h pre-hydrolysis and one pulse of 20 g/l glucan (2.0% w/v Avicel® or 3.3% w/v wheat straw) at day 4. In both feeding strategies, a total of 80 g/l glucan was fed into the system. Commercial cellulolytic cocktail (Celluclast 1.5 L® and Novozyme 188®), at low enzyme dosage and prior dialysis, and *P. antarctica* were used as biocatalysts.

Both strategies generated, after the pre-hydrolysis step at 50°C, glucose concentrations above 20 g/l either from Avicel® or pretreated wheat straw, with slightly higher concentrations in strategy 2 (initial 6.0% w/v Avicel® or 9.9% w/v wheat straw), due to higher initial glucan content (Figure 5). However, feeding strategy 1 generated higher MEL titres (Figure 5). Starting with lower glucan content (40 g/l), the stepwise feeding (2×20 g/l glucan) revealed to be more efficient in MEL production, probably because it maintained lower solid concentration throughout the process with beneficial rheological properties for enzymatic hydrolysis and/or fermentation (Figure 5). This effect was more pronounced when using wheat straw at the same glucan content (Figure 5b). The higher glucose concentration throughout the wheat straw conversion processes (both strategies 1 and 2) indicated that glucose consumption rate by *P. antarctica* was reduced in the presence of a complex substrate. The presence of the non-fermentable compounds (e.g. lignin), potential microbial inhibitors and/or lower oxygen availability due to the higher viscosity are possible explanations for this observation.

MEL production either from Avicel® or pretreated wheat straw at higher initial solids loading (feeding strategy 2) was similar to those obtained under simple SHF, reaching only 4.0 and 1.4 g/l, respectively (see Table 4). However, the lower initial solids with two-pulse feeding (strategy 1) resulted in increased MEL production both from Avicel^®^ (4.5 g/l) and wheat straw (2.5 g/l).

*Pseudozyma antarctica* T-34 was reported to produce 12 g/l of MEL from D-glucose with a fed-batch strategy requiring 280 g/l D-glucose and 21 days (yield of 0.04 g/g and productivity of 24 mg/l/h) [16]. In this study with *P. antarctica* PYCC 5048^T^, a lower MEL productivity was observed either from Avicel® (approx. 13 mg/l/h) or from pretreated wheat straw (7 mg/l/h). However, a slightly higher MEL yield with Avicel® (0.06 g/g) and similar MEL yield with pretreated wheat straw (0.03 g/g) were obtained when compared with those reached with *P. antarctica* T-34 [16].

This significant increase in MEL titre from pretreated wheat straw, maintaining similar MEL yields when compared to SHF and SSF processes (from 40 g/l glucan), indicated that further process optimization can be performed to increase MEL production from cellulosic materials. Moreover, the simultaneous use of the hemicellulose fraction can be explored since *Pseudozyma* species are able to convert xylose into MEL at yields comparable to glucose conversion [19].

## Conclusions

The technical feasibility of the conversion of cellulosic materials into glycolipid biosurfactants, MEL, was demonstrated for the first time in the present study. *Pseudozyma antarctica* PYCC 5048^T^ and *P. aphidis* PYCC 5535^T^ were able to produce MEL from Avicel® and pretreated wheat straw under SHF, SSF or Fed-batch SSF. Dialysis of commercial enzyme cocktails had a positive impact in yeast metabolism, including in MEL production, and the performance of the processes were compatible with the use of low enzyme dosage. Using a Fed-batch SSF strategy (2 pulses) with pre-hydrolysis (for 48 h), *P. antarctica* PYCC 5048^T^ generated, after 14 days, MEL titres of 4.5 and 2.5 g/l, from Avicel® and pretreated wheat straw, respectively. The MEL yield of 0.03 g/g from wheat straw can be potentially increased by using the hemicellulosic sugars (e.g. xylose) [19], together with further process optimization.

The ability of using renewable sugar-based substrates may contribute to sustainable MEL production at two levels: i) substrate – if using inexpensive lignocellulosic residues with low environmental impact; ii) downstream – MEL recovery from sugar-based substrates is one-step liquid-liquid extraction process, while the use of oil-based substrates requires a solvent intensive and low-yield downstream process. The use of lignocellulosic materials for MEL production represents an increase in process complexity, with pretreatment and enzymatic hydrolysis representing the main operational costs.

However, the use of inexpensive lignocellulosic substrates associated to straightforward downstream processing from sugary broths is expected to have a great impact in the economy of MEL production for the biosurfactant market, inasmuch as low enzyme dosage is sufficient for good system performance.

## Materials and methods

### Yeast strains, maintenance and standard cultivation conditions

*Pseudozyma antarctica* PYCC 5048^T^ (CBS 5955) and *Pseudozyma aphidis* PYCC 5535^T^ (CBS 6821) were obtained from the Portuguese Yeast Culture Collection (PYCC), CREM, FCT/UNL, Portugal. Yeasts were cultivated for 3 days at 25°C on Yeast Malt Agar (YM–agar) medium (yeast extract, 3 g/l; malt extract, 3 g/l; peptone, 5 g/l; glucose, 10 g/l; agar, 20 g/l). Stock cultures were prepared by propagation of yeast cells in liquid medium as described below for the inoculum and stored (in 20% v/v glycerol aliquots) at −70°C for later use. Inoculum was prepared by incubation of stock cultures of *P. antarctica* or *P. aphidis* at 28°C, 140 rpm, for 48 h, in liquid medium containing glucose (40 g/l), NaNO_3_ (3 g/l), MgSO_4_ (0.3 g/l), KH_2_PO_4_ (0.3 g/l) and yeast extract (1 g/l).

The cultivation media, containing a carbon source (40 g/l glucose or glucan) and the supplements MgSO_4_ (0.3 g/l), KH_2_PO_4_ (0.3 g/l) and yeast extract (1 g/l), was inoculated with 10% v/v of inoculum culture and incubated at 28°C, 140 rpm, for 4–14 days. All experiments were carried out in duplicate.

### Raw material - pretreatment and characterization

Glucose and Avicel® cellulose (Avicel PH-101, Sigma-Aldrich) were directly used for SHF an SSF experiments. Wheat straw was hydrothermally pretreated following the conditions previously established elsewhere [39]. A 0.6-L stainless steel reactor vessel (Parr Instruments Company, USA) was fed with wheat straw and water at liquid/solid ratio of 7. The reactor was heated to reach a final temperature of 210°C (non-isothermal conditions), 250 Psi, at 150 rpm, in 30 minutes. The reactor was cooled to 100°C in a water-ice bath, in 1.5 minutes. The solid and liquid phases were separated by pressing (up to 200 kg/cm^2^) using a hydraulic press. The solids were washed with water, filtered through Whatman No. 41 filter paper and dried at 50°C for 48 h [39]. The solid fraction was characterized by means of a quantitative acid hydrolysis [40] to determine cellulose content prior to use.

### Enzymatic hydrolysis

Celluclast 1.5 L® (Novozymes, Denmark), a cellulase from *Trichoderma reesei* QM-9414 exhibiting 58.4 FPU/ml and 31.2 U (β-glucosidase)/ml at 50°C, and Novozyme 188® (Novozymes, Denmark), a β-glucosidase from *Aspergillus niger*, exhibiting 690.8 U (β-glucosidase)/ml and 1.3 FPU/ml at 50°C, were used. The hydrolysis of cellulosic substrates (40 g/l glucan content) was performed at four different enzyme loadings:

(i) High (H) - 5.25% v/v Celluclast 1.5 L® (30 and 76.6 FPU/g_glucan_, respectively at 28°C and 50°C), and 0.75% v/v Novozyme 188® (49.47 and 213.2 U (β-glucosidase)/g_glucan_, respectively at 28°C and 50°C);
(ii) Medium-high (MH) - 3.50% v/v Celluclast 1.5 L® (20 and 51.1 FPU/g_glucan_, respectively at 28°C and 50°C) and 0.50% v/v Novozyme 188® (32.9 and 142.1 U (β-glucosidase)/g_glucan_, respectively at 28°C and 50°C);
(iii) Medium (M) - 1.75% v/v Celluclast 1.5 L® (10 and 25.5 FPU/g_glucan_, respectively at 28°C and 50°C) and 0.25% v/v Novozyme 188® (16.6 and 71.1 U (β-glucosidase)/g_glucan_, respectively at 28°C and 50°C);
(iv) Low (L) - 0.58% v/v Celluclast 1.5 L® (3.6 and 8.5 FPU/g_glucan_, respectively at 28°C and 50°C) and 0.08% v/v Novozyme 188® (5.1 and 22.1 U (β-glucosidase)/g_glucan_, respectively at 28°C and 50°C).

When applied in SSF or SHF, unless otherwise stated, the enzyme formulation containing Celluclast 1.5 L® and Novozyme 188® was previously dialysed (Spectra/Por, Spectrum®, MWCO: 12–14000) for 20 h, at 4°C, in 0.05 M potassium phthalate, pH 5.5.

Enzymatic hydrolysis studies were carried out in 250 ml-Erlenmeyer flasks, with a total working volume of 50 ml, containing Avicel® cellulose (4.0% w/v) or the solid fraction of pretreated wheat straw (6.6% w/v) suspended in 0.05 M potassium phthalate buffer pH 5.5. After incubation at 28°C or 50°C, 140 rpm, in presence of sodium azide (0.08% w/v), samples were taken periodically, centrifuged and the supernatants analysed for sugar composition and enzyme activity.

### Simultaneous saccharification and fermentation (SSF), Separate hydrolysis and fermentation (SHF) and Fed-batch SSF with pre-hydrolysis

Experiments were carried out in 250 ml Erlenmeyer flasks, with a total volume of 50 ml, containing: (a) cellulosic material - either Avicel® or the solid fraction of pretreated wheat straw at 4.0 and 6.6% w/v solids, respectively (both at approx. 40 g/l glucan); (b) cellulolytic enzyme cocktails in 0.05 M potassium phthalate buffer pH 5.5: either medium (M) dosage (1.75% v/v Celluclast 1.5 L® and 0.25% v/v Novozyme 188®) or low (L) dosage (0.58% v/v Celluclast 1.5 L® and 0.08% v/v Novozyme 188®); (c) inoculum 10% v/v, for initial yeast concentration of 0.5 g(CDW)/l; and (d) supplements containing MgSO_4_ (0.3 g/l), KH_2_PO_4_ (0.3 g/l) and yeast extract (1 g/l).

Several cultivation strategies were studied: (i) SSF experiments were started by simultaneous loading of both biocatalysts (yeast and enzyme). The flasks were incubated at 28°C, at 140 rpm for 10 days; (ii) SHF experiments were carried out under the same conditions of SSF, except that yeast loading and supplementation were performed only after 48 h of enzymatic hydrolysis at 50°C; (iii) Fed-batch SSF with pre-hydrolysis experiments with Avicel® and wheat straw were performed under the same conditions used in SSF and SHF concerning enzyme loading, medium and inoculum (always considered at day 0) with the following modifications: (iii.a) Feeding strategy 1, 40 g/l glucan content (4.0% w/v Avicel® or 6.6% w/v wheat straw) were initially fed for pre-hydrolysis (48 h at 50°C) and two additional pulses of 20 g/l glucan were fed (2.0% w/v Avicel® or 3.3% w/v wheat straw) at day 0 and 4; (iii.b) Feeding strategy 2, 60 g/l of glucan content (6.0% w/v Avicel® or 9.9% w/v wheat straw) were initially fed for pre-hydrolysis (48 h at 50°C) and an additional pulse of 20 g/l glucan was fed (2.0% w/v Avicel^®^ or 3.3% w/v wheat straw) at day 4.

### Analytical methods

#### Yeast growth and viability

Cell growth was followed spectrophotometrically [optical density (OD) at 640 nm], by quantification of cell dry weight (CDW) and/or by quantification of viable yeast cells (colony forming units – CFU). CDW was determined with 1 ml of culture broth. Culture broth was centrifuged at 13000 rpm for 10 min, the pellet was washed twice in deionized water and dried at 100°C for 24 h. Viable yeast cells were determined, after 96 h culture, by platting 100 μL of appropriated dilution in YM-agar and incubation at 25°C, for 48 h, for determination of CFU.

#### Sugar profile

Supernatants were collected, filtered through a 0.45 μm-pore-size filter and analysed for glucose, xylose and erythritol quantification in high performance liquid chromatography (HPLC) system (Merck Hitachi, Darmstadt, Germany) equipped with a refractive index detector (L-7490, Merck Hitachi, Darmstadt, Germany) and an Aminex HPX-87H column (300 mm × 7.8 mm, Bio-Rad), at 50°C. Sulfuric acid (0.005 M) was used as mobile phase at 0.4 ml/min.

#### Cellulolytic enzyme assays

Cellulase activity was assessed according to Ghose [41], as filter paper activity (FPase) by measuring the release of reducing sugars from Whatman number 1 filter paper. Reducing sugars were estimated by the dinitrosalicylic acid (DNS) method [42]. The assay was modified according to King et al. [43] by using filter paper cylinders (2 × 2.2 mg), in potassium phthalate buffer (0.05 M, pH 5.5), at 28°C or 50°C, in a total reaction volume of 125 μl. Filter Paper Unit (FPU) is defined as the amount of enzyme required to release 1 μmol of glucose reducing equivalent per minute, under the conditions defined by Ghose [41].

β-Glucosidase activity was assayed in a reaction mixture (0.3 ml) containing 5 mM p-nitrophenyl-β-D-glucoside (pNPG, Sigma, USA), 0.05 M potassium phthalate, pH 5.5, and appropriately diluted enzyme solution. After incubation at 50°C for 60 min, 0.15 ml of 1 M Na_2_CO_3_ was added to stop the reaction [44]. The p-nitrophenol absorbance (pNP) was measured at 405 nm. One unit (U) of β-glucosidase activity is defined as the amount of enzyme releasing 1 μmol pNP per minute.

#### MEL and fatty acids profile

The total fatty-acid pattern of biological samples was determined by methanolysis of freeze-dried culture broth [19,45]. The resulting reaction was extracted with hexane (1 ml) and 1 μl of the organic phase was analysed by gas chromatography to determined fatty acid concentrations against C7:0 internal control/standard [19].

MELs were quantified through the amount of C8, C10 and C12 fatty acids [19]. The identification of MEL was confirmed by thin layer chromatography (TLC) after liquid-liquid extraction of the culture broth with one volume of ethyl acetate [19]. MEL (MEL-A, -B, -C and -D) produced by *P. aphidis* cultivation on soybean oil, was used as reference [19].

#### Statistical analysis

Statistics were performed by analysis of variance (ANOVA) and p-values of the differences between groups were corrected for simultaneous hypothesis testing according to Tukey’s method. The level of significance was set at p < 0.05.

## Abbreviations

MEL: Mannosylerythritol lipid
SHF: Separate hydrolysis and fermentation
SSF: Simultaneous saccharification and fermentation
FPU: Filter paper unit
PYCC: Portuguese yeast culture collection
CDW: Cell dry weight
